# Community structure of rare methanogenic archaea: insight from a single functional group

**DOI:** 10.1093/femsec/fix126

**Published:** 2017-10-03

**Authors:** Sizhong Yang, Matthias Winkel, Dirk Wagner, Susanne Liebner

**Affiliations:** 1 GFZ German Research Center for Geosciences, Helmholtz Centre Potsdam, Section 5.3 Geomicrobiology, 14473 Potsdam, Germany; 2 State Key Laboratory of Frozen Soil Engineering, Northwest Institute of Eco-Environment and Resources, Chinese Academy of Sciences, 730000 Lanzhou, China

**Keywords:** rare biosphere, methanogenic archaea, conditionally rare taxa, species richness, beta diversity

## Abstract

The rare biosphere, the low abundant microbial populations, is suggested to be a conserved way of microbial life. Here we conducted a molecular survey of rare methanogenic archaea in the environment targeting the *mcrA* gene in order to test if general concepts associated with the structure of the rare bacterial biosphere also apply to single functional groups. Similar to what is known about rare bacterial communities, the contribution of rare methanogens to the alpha diversity is much larger than to Bray-Curtis measures. Moreover, a similar core group of methanogens harbored by the abundant and rare communities suggests similar sources and environmental controls of both groups. Among the communities of different levels of rarity, the conditionally rare methanogenic taxa largely account for the overall community dynamics of the rare biosphere and likely enter the dominant community under favorable environmental conditions. In addition, we observed a positive correlation between the alpha diversity and the production of methane when the rare taxa were taken into account. This supports the concept that increasing microbial biodiversity enhances ecological function. The composition and environmental associations of the rare methanogenic biosphere allow us to conclude that rarity is a conserved way also for single functional groups.

## Teaser

Similar to bacteria, rarity is a conserved way of life also for methanogenic archaea.

## INTRODUCTION

In microbial communities, it is common that a tiny fraction of abundant species which account for the majority of the observed community coexists with a large number of rare taxa represented by very few individuals (Pedros-Alio 2012). The concept of the rare biosphere was proposed to describe the microbial populations representing 0.1%–1% of the microbial community (Sogin *et al.*^2006^; Reid and Buckley 2011). The rare taxa could hardly be detected by first-generation sequencing techniques (Pedros-Alio 2012; Lynch and Neufeld 2015), but in recent years the advance and extensive application of next-generation sequencing (NGS) has provided a great microscope to bring them into focus (Pedros-Alio 2007; Lynch and Neufeld 2015). With consistent detection of a wide range of rare microbial species in the soil, ocean and our own bodies, rarity is considered to be a conserved way of microbial life (Lynch and Neufeld 2015).

The rare biosphere is overall important for the local species richness (alpha diversity) but is less significant for the abundance-based beta diversity (Sogin *et al.*^2006^; Pedros-Alio 2012; Beck, Holloway and Schwanghart 2013). Recent numeric evaluations suggest that the rare biosphere can explain microbial community dynamics to some extent (Shade *et al.*^2014^; Coveley, Elshahed and Youssef 2015). Some members in the rare biosphere can play disproportional roles in microbial networks and metabolic processes in the environment (Pester *et al.*^2010^; Hugoni *et al.*^2013^; Lynch and Neufeld 2015). In addition, the broadly distributed rare taxa are also subjected to environmental filtering in a suite of ecological conditions (Caron and Countway 2009; Lynch, Bartram and Neufeld 2012; Pedros-Alio 2012; Lynch and Neufeld 2015). Minor changes in environmental conditions can result in rapid and frequent reassemblies of the taxonomic composition of the rare communities (Caron and Countway 2009; Hugoni *et al.*^2013^; Vergin *et al.*^2013^), and some rare species could bloom in response to changing environmental conditions (Reid and Buckley 2011; Pedros-Alio 2012; Vergin *et al.*^2013^). These results indicate that environmental forces on the rare taxa exist.

The community dynamics within the rare biosphere reflect different subsets of rarity. The conditionally rare taxa (CRT) can become abundant when provided with optimal growth conditions for instance in the course of climate change. In contrast, some taxa remain persistently low in abundance because they grow slowly or survive in dormant stage (Sogin *et al.*^2006^; Lynch and Neufeld 2015). Furthermore, the large variability of different levels of rarity may have practical implications and suggest potential roles in metabolism and ecological functions. Some rare taxa are responsible for keystone physiological functions, e.g. involved in element cycling (Hewson *et al.*^2009^; Pester *et al.*^2010^) or in bioremediation (Kleindienst *et al.*^2016^), while the majority of the rare biosphere is regarded as a genetic seed bank (Sogin *et al.*^2006^; Pedros-Alio 2012). Therefore, the different fractions of the rare biosphere form a comprehensive backup system to maintain ecosystem resilience. Despite this, the relevance of the rare biosphere to the total community was often ignored and in many cases the rare taxa were removed from the analysis at a specific numerical or ecological threshold (Lynch and Neufeld 2015).

Our current knowledge about the rare biosphere is largely derived from environmental surveys targeting the bacterial 16S rRNA gene (Sogin *et al.*^2006^; Galand *et al.*^2009^; Youssef, Couger and Elshahed 2010; Pedros-Alio 2012; Shade *et al.*^2014^; Lynch and Neufeld 2015). This means that most studies on the rare biosphere describe microbial members with a large redundancy in their functional repertoire. The rare biosphere of a single functional guild is, however, poorly characterized. Pester *et al.* (2010) used stable isotope probing to focus on sulfate reducers in peatlands and found a rare bacterium accounting for only 0.006% of the 16S rRNA gene abundance but playing a pivotal role in sulfate reduction. To the best of our knowledge, there is no study of the rare biosphere based on functional gene markers yet. Since the dynamic and ecological meaning of the rare biosphere are not well known, an exploration of similarities and dissimilarities of the rare biosphere between the overall microbial community and single functional guilds will improve our current understanding on rarity as a conserved way of life.

As an example for a single functional guild, we picked methanogenic archaea (methanogens). Methanogens are responsible for the production of methane, a very potent greenhouse gas in a variety of global environments and were frequently surveyed in environmental samples by using the functional *mcrA* gene marker (Luton *et al.*^2002^; Frank-Fahle *et al.*^2014^; Liebner *et al.*^2015^; Wen *et al.*^2017^; Yang *et al.*^2017^). This single gene serves both as a phylogenetic and functional marker gene. We hypothesize that the same concept on rarity as a conserved way of life that are proposed for overall bacteria also apply for the single functional group of methanogenic archaea, it means that the community patterns of the rare methanogenic archaea are in line with what we know from the rare bacterial biosphere. We, therefore, used 454 pyrosequencing to compare the taxonomic composition of rare and abundant methanogens at different arbitrary thresholds of rarity. We also calculated the contribution of rare methanogens to alpha- and beta diversity measures. In addition, we made an exploratory analysis on the significance of the rare biosphere for the process of methane production.

## MATERIAL AND METHODS

### Study site and sampling

Soil samples were collected from four different sites, two alpine swamps and two alpine meadows on the northeastern Tibetan Plateau in August 2012. The two swamps are the Donggi Cona Lake region (DCL) and Haibei Station (HAI), while the meadows are Huashixia (HUA) and Gande (GAN). The swamps and meadows are influenced by discontinuous permafrost and seasonally frozen ground, respectively. From these sites, a total of 16 samples were taken at the following depths: DCL: 5, 20, 35, 50 and 65 cm; GAN: 5, 20, 35, 70, 100 and 150 cm; HAI: 5, 20, 40 and 60 cm; and HUA: 5, 15, 35 and 55 cm. Full description of the study sites and their location are available in a recent publication (Yang *et al.*^2017^).

### DNA extraction and *mcrA* 454 amplicon sequencing

The DNA was extracted in triplicates on-site from 0.5 g of fresh soil with the FastDNA SPIN kit (MP Biomedicals, Germany) and then purified with the MiniElute PCR Purification Kit (Qiagen, Germany). Prior to this amplicon pyrosequencing, a pilot shotgun sequencing has combined the upper (top) and lower (down) horizons of HAI and HUA (HAI_t: 5 + 20 cm, HAI_d: 40 + 60 cm; HUA_t: 5 + 15 cm, HUA_d: 35 + 55 cm). The pooled DNA templates were inherited in this study.

The *mcrA* gene was amplified with the barcoded primer set of mlas and mcrA-rev (Steinberg and Regan 2008). PCR reactions were performed in 50 μl solutions with 1.0 μl of template DNA and 0.1 μM of primers. The PCR amplification started with an initial denaturation at 95°C for 3 min, followed by 30 cycles at 94°C for 30 s, 55°C for 45 s, and 72°C for 45 s, with a final extension at 72°C for 5 min. Afterwards, the triplicate PCR products for each sample were pooled and purified with the MiniElute PCR purification kit (Qiagen). Pyrosequencing of the equimolar PCR product pool was implemented on a Roche GS-FLX + + Titanium platform at Eurofins (Germany).

### Meta-data processing

The mothur software (Schloss *et al.*^2009^) was employed to process the raw data and to assign the operational taxonomic unit (OTU). The raw 454 sequence data were first allocated by samples according to barcodes. To remove sequences with translation frameshift errors, the fasta-format sequences were filtered with the FrameBot tool available at https://github.com/rdpstaff/Framebot (Wang *et al.*^2013^). The sequences were further screened to reject those with errors in matching barcodes or primers, homopolymer stretch over 6 nucleotides, ambiguous bases or average quality scores less than 25. We also discarded sequences shorter than 300 or longer than 600 nucleotides. The sequences were then aligned against pre-aligned *mcrA* sequences which were provided by the FunGene Pipeline (http://fungene.cme.msu.edu/) (Fish *et al.*^2013^). After removing the bad alignments, the remaining sequences were subjected to chimera detection with the UCHIME algorithm (Edgar *et al.*^2011^) in mothur. Using the furthest neighbor clustering method, the valid sequences were assigned into OTUs at a cutoff of 0.16 for *mcrA* nucleotide sequences which corresponds to 3% dissimilarity (97% similarity) of the 16S rRNA gene (Yang *et al.*^2014^).

We obtained technical duplicates for each sample and the subsequent Non-metric multidimensional scaling (NMDs) analysis showed good congruency of the community patterns. Thus, we combined the data prior to further sequence analysis (see Yang *et al.*^2017^ for details). The abundance-based coverage estimator Chao1 and Shannon, rarefaction curves and beta diversity metrics were calculated or visualized with *R* packages of vegan (v.2.0–7) (Oksanen *et al.*^2013^) and phyloseq (v.1.10.0) (McMurdie and Holmes 2013). The abundance differentiation over samples was visualized with ggplot2 v.1.0.0 (Wickham 2009). The taxonomy for the rare taxa was first classified in mothur against a reference database (Yang *et al.*^2014^). For the ambiguous ones, the representative sequences were further classified in ARB by referring to an updated reference ARB database (Angel, Claus and Conrad 2012).

### Classification of different rarity thresholds

Currently, the rare biosphere is arbitrarily defined in the threshold window of 0.1%–1% (Reid and Buckley 2011; Lynch and Neufeld 2015). This study defines the abundant and rare taxa as the OTUs having an average relative abundance above and below 1% across all samples. In addition, we followed the concept of ‘conditionally rare’ which means microbial taxa that are typically in low abundance in one locality but occasionally become prevalent in at least one other sample (Shade *et al.*^2014^; Lynch and Neufeld 2015). Thus, the abundance of a CRT exhibits large skewness of the data distribution. From a technical perspective, the skewness in this study was examined by the fluctuation of the relative abundance of methanogens across samples. The CRT was assigned if an OTU exhibits a maximum relative abundance at least 100 times higher than its minimum value (max:min > 100). If the ratio is less than 5, that is the abundance rarely fluctuates among samples, it will be grouped into the ’permanently rare taxa (PER)’. If the ratio ranges in between, we assign it into ‘RARE’.

### Contribution of rare taxa to the total beta diversity measure

The contribution of the rare taxa to the beta diversity was examined by partitioning the Bray-Curtis (BC) distance matrix, an abundance-based metrics, according to the calculation function below (Oksanen *et al.*^2013^).

\begin{equation*}
B{C_{jk}} = \frac{{\sum \left| {{X_{ij}} - {X_{ik}}} \right|}}{{\sum \left( {{X_{ij}} + {X_{ik}}} \right)}}
\end{equation*}

where *j* and *k* are communities; *BC* is the Bray-Curtis dissimilarity between communities *j* and *k*; *X* is the relative abundance of taxon *i*. The BC dissimilarity matrix is a scaled summation of abundance differences between two communities and can be partitioned for a subset population from the community (Shade *et al.*^2014^). The BC measure was partitioned by using a custom *R* script which puts the summation of the CRT taxa in the numerator of the BC dissimilarity expression but uses all of the taxa when calculating the scaling summation in the denominator. Afterwards, the BC dissimilarity of CRT was divided by the total community BC dissimilarity to report the contribution of the CRT to the total beta diversity. Then we compared the contribution of different groups of rare taxa to the total beta diversity between the abundant and rare biosphere, and among different groups among the rare taxa. The percentage to the total BC metrics is demonstrated as boxplots with quantile summaries.

### Robustness of rare biosphere dendrogram

To investigate the response of the community structures to increasing rareness, we carried out a hierarchical clustering analysis on a series of subsets of rare taxa. Briefly, the rare taxa data were subset according to a gradient of lowering thresholds of rarity from <1% down to 0.1% by decrement of 0.05%. The consequent 19 hierarchical clusters were then converted into dendrogram objects by using the *R* package of dendextend (Galili 2015). The similarity of dendrograms was subsequently compared in terms of the Baker's Gamma Index (BGI) with the function ‘cor_bakers_gamma’ from the dendextend package. The BGI (also known as Goodman and Kruskal's gamma) is a measure of association (similarity) between two hierarchical clustering dendrograms (Baker 1974). The value can range between –1 and 1. With near 0 values meaning that the two trees are not statistically similar, while index −1 and 1 represent 100% negative association (or perfect inversion) and 100% positive association (or perfect agreement), respectively. Finally, the pairwise BGI matrix was visualized by the *R* corrplot package (v0.77) (Wei and Simko 2016).

### Data deposition

The *mcrA* gene sequence data were deposited in the NCBI Sequence Read Archive with the submission ID SRP046048.

## RESULTS

### Alpha diversity and composition of rare methanogenic communities

The sequencing yielded 187 789 raw sequences, and 163 644 reads passed the quality filtration. Most samples contained more than 5000 valid sequences, except for GAN1 and GAN2 with 1119 and 2702 reads, respectively (also see Fig. S1, Supporting Information). As the sequence amounts largely varied, subsampling would waste the massive sequencing effort and exclude potential rare members. Moreover, subsampling can cause secondary bias (McMurdie and Holmes 2014). The valid dataset was therefore not subsampled in this study. The sequences were assigned into a total of 175 OTUs (including 40 singletons). Among them, 25 OTUs are identified as abundant taxa with average relative abundance > 1%. Altogether they account for 85% to the total amount of reads but a minor proportion (14.3%) to the overall diversity. The abundant OTUs are dominated by members from *Methanoregula*, *Methanomassiliicoccus*, *Methanosaeta* and *Methanosarcina* (Table S1, Supporting Information, shows the average relative abundance of each genus). The detailed data about the abundant taxa were reported elsewhere (Yang *et al.*^2017^).

The 150 rare OTUs contributed 85.7% to the total OTU diversity but only 15% to the total OTU counts. Among the rare population, 18 OTUs were identified as CRT, with a mean abundance of 8.1% in the total community. Another 84 permanently rare taxa (PER) only represented a very small fraction (0.25%) of the total abundance but a contrasting high fraction (48%) to the total OTU richness. The PER group also include the 40 singletons. The remaining 48 OTUs were assigned as ‘RARE’ with an average abundance of 5.4% (Table S2, Fig. S2, Supporting Information). In addition, the three rare clusters exhibited a large variability in the abundance among the clusters; and each cluster also shows a different extent of abundance fluctuation across the samples. The CRT has an overall high but fluctuating abundance distribution across the samples, while the PER showed a flat variation across samples (Fig. S2).

**Figure 1.**
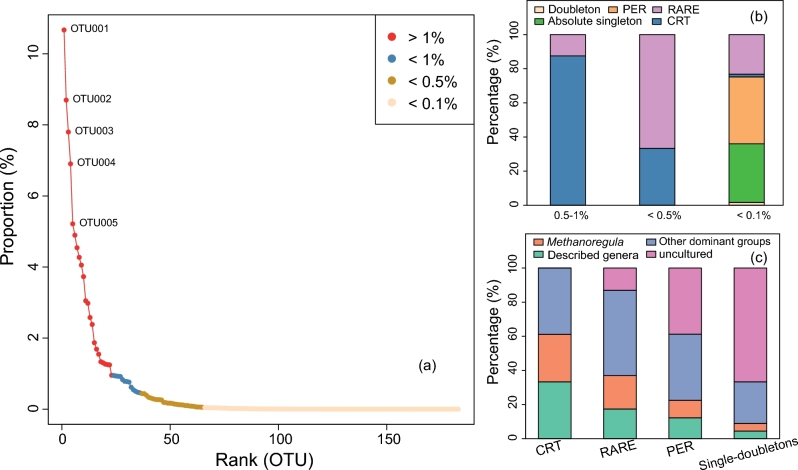
(**a**) Rank abundance curve with different thresholds of mean relative abundance, (**b**) relative abundance of the different classes of rarity compared to the total community at a given threshold of relative abundance and (**c**) taxonomic composition of the classes of rarity. The ‘CRT’ and ‘PER’ denote ‘conditionally rare taxa’ and ‘permanently rare taxa’, respectively. All the other rare ones were assigned as ‘RARE’. ‘Doubleton’ means the OTUs with two sequences occurring in one or two samples, and the ‘Absolute singleton’ denotes the OTUs with sequence amount of 1 and only occurring in one sample.

Among the rare biosphere, *Methanoregula* is the most abundant group. Besides, four other taxa (*Methanobacterium*, *Methanosaeta*, *Methanosarcina* and *Methanthermobacter*) appear to be very dominant and are grouped as ‘other dominant groups’ (Fig. S3, Supporting Information). Other sequences that could be assigned to the described taxa such as *Methanoflorens*, *Methanomassiliicoccus* and *Methanomethylovorans* (Fig. S3) are grouped as ‘described genera’, while the remaining taxa are grouped as ‘uncultured’ (Fig. 1). In the rank abundance curve, the CRT roughly fall in the <1%, while the PER are mainly mapped in the tail (Fig. 1). Along with the changing rarity thresholds from <1% to <0.5% then to 0.1% (Fig. 1a), i.e. towards the end of the rank-abundance tail, the fraction of CRT decreased in the rare biosphere while the permanently rare OTUs, doubletons and singletons increased, illustrating that the CRT are not among the most rare OTUs (Fig. 1b). In addition, the abundance of *Methanoregula*-related OTUs and other described groups decreased whereas the yet uncultured groups increased with increasing rarity (Fig. 1c).

**Figure 2.**
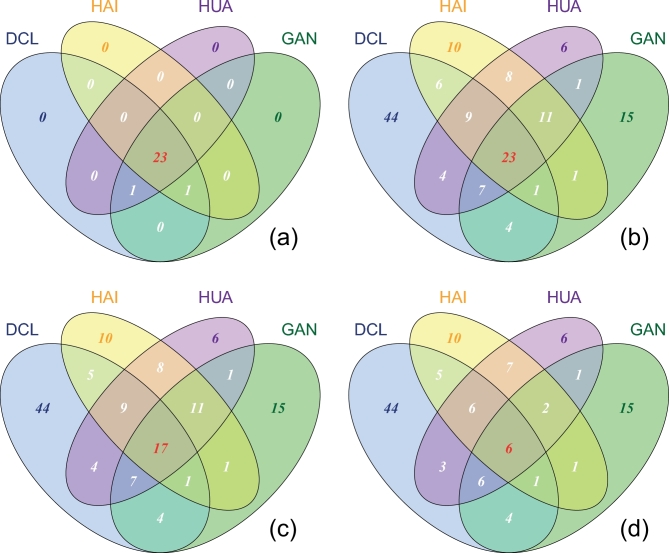
Venn diagram showing the shared and specific OTUs for the abundant taxa with the mean relative abundance higher than 1% across samples (**a**), and for the rare taxa with increasing rarity by lowering the threshold of average relative abundance from <1% (**b**), to 0.5% (**c**) and down to 0.1% (**d**). DCL: Donggi Cona Lake region; HAI: Haibei Station; HUA: Huashixia and GAN: Gande.

The composition of shared (widely distributed over many samples) and specific (indigenous) rare OTU phylotypes was displayed with Venn diagrams in order to compare the different components between the abundant and rare biosphere, and among the rare population. The abundant taxa are almost entirely shared in a core assemblage (23 OTUs) of methanogenic archaea among sites (Fig. 2a, see Yang *et al.*^2017^ for details). In contrast, the rare biosphere constitutes a larger fraction of locally specific (indigenous) than shared OTUs. Moreover, among the rare biosphere the taxa which were completely or partially shared by different sites decreased with increasing level of rarity, whereas the OTUs shared by two sites slightly decreased in a few cases. The indigenous OTUs stayed constant in all sampling sites, pointing towards really low abundant OTUs (Fig. 2b–d). This observed effect is not due to undersampling as the rarefaction curves in all samples have reached plateaus (Fig. S1). Thus, the contrast between the indigenous and shared rare taxa became greater, which implies that the increased site specificity across sites is associated with the rare population.

The alpha diversity was highest in the sites where *mcrA* gene copy numbers and potential methane production were also highest (Yang *et al.*^2017^). We correlated the alpha diversity indices with methane production rates and *mcrA* gene copies of each sample (Table 1). Among the three alpha diversity indices, Chao1 and ACE reflect the species richness, while the Shannon index depends on both species richness and evenness. For the abundant taxa, none of the three indices were observed to statistically correlate with the gene copy numbers or methane production rates. In contrast, when the rare taxa are taken into account, this correlation is statistically significant for Chao1 and ACE. The correlation coefficient and to a certain extent the significance even slightly increased when only considering the rare biosphere. However, there is no correlation between gene abundance or methane production rates and the Shannon index for either the dominant or the rare biosphere.

**Table 1.** Correlations between the diversity indices and functional activity by means of methane production rate and the *mcrA* gene copies.

Biosphere	Indices	Copies	Rate_Ac	Rate_H_2_/CO_2_
All	Chao1	0.64**	0.56**	0.53*
	ACE	0.67***	0.59**	0.56**
	Shannon	0.092	0.14	0.22
Abundant	Chao1	0.035	-0.018	0.039
	ACE	0.054	0.013	0.085
	Shannon	–0.018	0.031	0.12
Rare	Chao1	0.70***	0.63**	0.58**
	ACE	0.76***	0.67***	0.61***
	Shannon	0.11	0.17	0.29

For the original values of methane production rate and *mcrA* gene copies, refer to Yang *et al.* (2017). Rate_Ac and Rate_H_2_/CO_2_ stand for the methane production potential rates with substrates of acetate for acetoclastic methanogenesis and H_2_/CO_2_ for hydrogenotrophic methanogenesis, respectively. The Pearson correlation significances of P < 0.01, 0.05 and 0.1 are indicated by ‘***’, ‘**’ and ‘*’, respectively.

### Contribution of rare taxa to beta diversity measures

**Figure 3.**
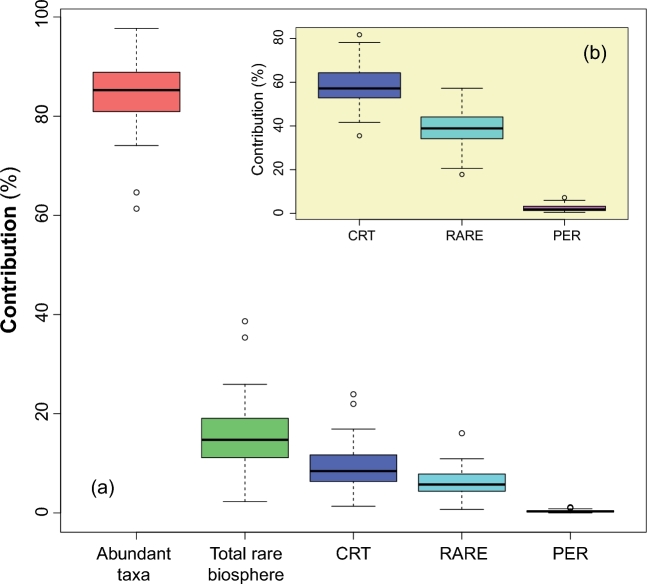
Boxplot showing quantile summary of the proportional contribution of different groups of rare taxa to the total Bray-Curtis distance matrices of the total methanogenic archaeal community (**a**) and of the rare biosphere in the inlet plot (**b**). The ‘CRT’ and ‘PER’ represent the ‘conditionally rare taxa’ and ‘permanently rare taxa’, respectively. And ‘RARE’ is composed of all the other rare ones in the rare biosphere.

Regarding the total BC dissimilarity, the abundant taxa account for about 84.5% to the total differentiation across communities (Fig. 3a). On the other hand, the rare biosphere including the CRT, RARE and PER collectively accounts for a minor proportion (15.5%) to the total. In some individual samples, however, the rare biosphere is an important component, representing up to 35.6% of total community variation (Fig. 3a, Table S3). The CRT, as the paramount component among the rare biosphere, contributed 1.3–24.7% (8.6% on average) to the total community. Moreover, the CRT alone contributed 58.8% to the beta dissimilarity in the rare biosphere (Fig. 3b). In contrast, the most diverse PER group only makes up a tiny proportion of the total BC matrix of the rare community (Table S3).

**Figure 4.**
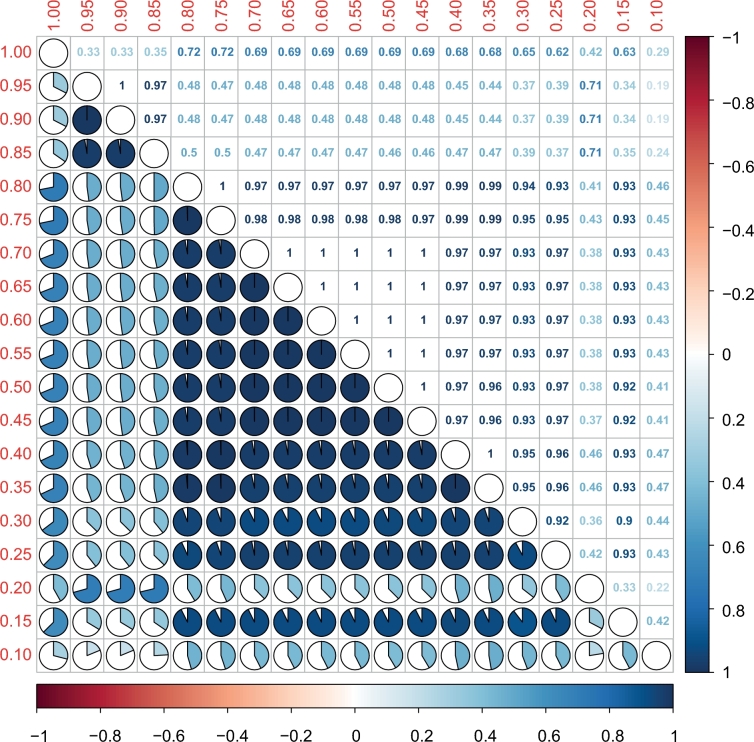
Baker's Gamma Index between pairs of dendrograms which indicates the structural similarity between two hierarchical clustering (dendrograms). The value which is closer to 1 means that the two trees are more statistically similar. Dendrograms are based on Bray-Curtis distance matrices and reflect subsets of rare populations calculated for different thresholds of rarity. The red values along the left and upper axes show the upper threshold of each subset. The lower triangular pie plots show how similar the topography of the dendrograms is. The upper triangular part gives the according numerical values. The color bar denotes the range of Baker's Gamma Indices.

To assess the influence of rarity on the community structure of the rare biosphere, we examined the robustness of relative positions of each branch in the hierarchical dendrograms with a gradient of putative rarity by using the BGI matrix. According to the correlation plot, it appears that excluding some OTUs from the rare population, for instance through adjusting the rare threshold from 0.95% to 0.90%, does not lead to significant change in the topographical structure of the dendrogram. However, removing some rare phylotypes caused visible differences along the rare threshold spectrum from 0.90% to 0.80%, 0.80% to 0.20% and the transition from 0.2% to 0.15% (Fig. 4), which is generally owing to the members of CRT and RARE. The interruptions of the similarity imply that the dendrogram structure (i.e. community structure) is not linearly related to each member of the rare biosphere. Excluding some rare taxa will result in substantial change in rare community structure.

## DISCUSSION

Following the pioneering work on the rare biosphere in 2006 (Sogin *et al.*^2006^), the rare biosphere has been increasingly explored within complex microbial communities in the environment (Pedros-Alio 2007; Galand *et al.*^2009^; Pester *et al.*^2010^; Gobet *et al.*^2012^; Kleindienst *et al.*^2016^). Despite the increasing awareness of rare bacterial communities, there is a lack of information and comparative approaches on the rare biosphere of single functional guilds. In this study, we revealed that rarity is a conserved way of life also for single functional groups through a case study on the composition, structure and fractions of rare methanogens. Analysis of the environmental factors showed similar underlain controls as for the dominant methanogens (data not shown). Additionally, including the rare methanogenic biosphere into alpha diversity analysis revealed positive correlations with methane production rates and methanogenic abundance.

The rare methanogens harbor a core assortment whose elements correspond to the dominant population, suggesting a positive influence of abundant taxa on the structure of the rare ones. The major players such as the most commonly identified taxon *Methanoregula* are the same within both the abundant and the rare biospheres. Similar occurrence of core members in both rare and abundant populations was identified in other environmental samples (Galand *et al.*^2009^; Logares *et al.*^2013^; Coveley, Elshahed and Youssef 2015). Such consistency is possibly due to the high number of abundant species that allow for a higher likelihood of occupying a large number of niches (Nazaries *et al.*^2013^). There is evidence from Arctic marine microbial communities showing that the abundant taxa directly influence the size and composition of the rare biosphere (Galand *et al.*^2009^). The core assemblage may also lay a foundation for the biological interaction and mutual exchange, i.e. feedback (pool/source) from abundant taxa and recruitment from the rare biosphere. This part of the rare biosphere may possibly act as the active backup system that readily responds to environmental disturbances.

The rare community also contained a range of indigenous rare methanogens varying between the samples (Fig. 2). Indigenous rare taxa were also found in marine and soil bacterial communities (Pommier *et al.*^2007^; Galand *et al.*^2009^; Gobet *et al.*^2012^). In a microbial habitat, the subtle difference within the microenvironments can generate a variety of niches and a range of local environmental stresses (Souza *et al.*^2008^; Szekely and Langenheder 2014). The isolation and natural selection play important roles in the diversity and development of local endemism (Souza *et al.*^2008^), imposing selection pressures on all levels of rare taxa (Hugoni *et al.*^2013^; Vergin *et al.*^2013^; Lynch and Neufeld 2015). The rare species will consequently be assembled and structured due to their sensitivity and affinities to the subtle ecosystem settings (Reid and Buckley 2011; Hugoni *et al.*^2013^; Vergin *et al.*^2013^). The low abundant microbes were thought to be more likely indigenous candidates (Hedlund and Staley 2004). Nonetheless, the indigenous rare species represent a very diverse genetic seed bank allowing for genetic resilience. We hypothesize that the permanently rare methanogenic taxa are not metabolically active but rather contribute to processes like horizontal gene transfer, i.e. acting like *mcrA* gene bearer for example. Furthermore, they may reflect an archive of historically abundant or CRT whose living conditions turned unfavorable at a given time in the past and may have turned into dormant state. Finally, we cannot completely rule out that some of the rare OTUs are the result of sequencing artifacts even though we applied thorough translational checks as described before.

Our results support that the rare taxa contribute overproportionally to the alpha diversity, in particular to the species richness, but underproportionally to the beta diversity measures (Sogin *et al.*^2006^; Pedros-Alio 2012; Bachy and Worden 2014; Bagchi *et al.*^2015^). Despite the stunning contribution to alpha diversity, the low abundance of rare methanogenic taxa explains their minor contribution to the beta diversity metrics which are highly dependent on the distribution of the most common taxa (Heip, Herman and Soetaert 1998; Beck, Holloway and Schwanghart 2013). The rare taxa were thus often excluded from analyzing community differentiation (Lynch and Neufeld 2015). However, that does not necessarily mean that the rare taxa are dispensable for individual communities. Our study demonstrates that the contribution of the rare methanogenic taxa to the total community Bray-Curtis dissimilarity varied greatly (2.2%–35.6%) across the samples. Thus, completely excluding the rare taxa from beta diversity analysis may blunt the interpretation of some data sets. In fact, a related study suggested that including the rare members improves the representation of the beta diversity (Youssef, Couger and Elshahed 2010). Hence, the rare biosphere is also an integral component of the whole community for single functional guilds. Of course, whether including or excluding the rare biosphere into numeric ecology analysis depends on the specific research questions.

For the rare methanogenic biosphere in our case study, the change in similarity with increasing level of rarity is not unimodal. Several jumps in community similarities at different rarity thresholds (Fig. 4) imply that some rare phylotypes are more important to the rare community composition. Among the three distinct groups of rarity, the CRT substantially varied in abundance across samples, which supports that the CRT is a continually changing assortment of taxa rather than a static collection of species that are always rare. The RARE group reacts like the CRT with exceptions in two samples. Different rare fractions were also identified in surface coastal waters (Hugoni *et al.*^2013^) and were considered to use different life strategies (Lynch and Neufeld 2015). The differentiation among the rare methanogenic biosphere allows concluding that even the rare biosphere of a single guild is not randomly assembled. Each subset of the rare biosphere probably represents different ecological potentials with regard to adaptation capacity, activity levels or growth rates.

The influence of rare taxa on the community variation is to some extent dependent on the rarity threshold. We have arbitrarily defined the rare biosphere according to previous studies using variable abundance thresholds (Galand *et al.*^2009^; Reid and Buckley 2011). These thresholds determine if a community member is ‘rare’ or ‘non-rare’. The CRT and some RARE members are sensitive to this cutoff assignment, which also directly influences beta diversity measures. For the NGS data, a reasonable trade-off on the rarity threshold could have practical importance regarding the diversity of both rare and abundant groups. In addition, we also found that with elevated level of rarity, the described genera decline while the undescribed taxa increase (Fig. 1c), which shows that the taxonomy of rare lineages remains largely unknown. Cultivation efforts are thus encouraged to discover novel phylogenetic diversity and update the current reference database so as to improve taxonomic identification of rare biosphere organisms. However, cultivating novel rare microorganisms of the dormant or inactive rare ones can be particularly challenging (Zengler *et al.*^2002^; Lynch and Neufeld 2015), and culture-independent approaches, e.g. genome assembling and analysis may provide complementary insights which could also be beneficial for the identification of rare species.

Finally, this study implies a positive relationship between microbial alpha diversity (Chao1 and ACE indices) and methane production activity when taking into account the rare taxa. Such a positive correlation was also reported for bacterial denitrifiers (Philippot *et al.*^2013^) or sulfate reducers (Pester *et al.*^2010^). This statistic correlation has been frequently observed in macroorganisms such as grasslands where increased diversity can improve the ecosystem productivity (McGrady-Steed, Harris and Morin 1997; Loreau and Hector 2001; Zavaleta and Hulvey 2007). The ecological rationality inherent to this positive influence of alpha diversity on functionality also appears applicable to microbial communities. In general, diverse communities are considered to utilize the available resources more exhaustively than simple communities because of a diversity effect for functional complementarity (Tilman 1999; Jousset *et al.*^2011^). And some rare taxa have been proved to act as keystone species disproportionally contributing to biogeochemical processes (Giovannoni and Stingl 2005; Pester *et al.*^2010^). Therefore, including the rare taxa into analysis may have practical feasibility in the way that it reveals a positive correlation between alpha diversity and function. However, the observed correlation needs careful verification and more evidences.

## CONCLUSION

The present study revealed that the rare methanogenic species constitute an indispensable part of methanogenic communities, potentially being an important basis to ensure ecological resilience in response to various environmental stresses for single functional groups. Similar to what we know about rare bacteria, the rare biosphere of methanogenic archaea contributed overproportionally to the overall OTU richness but poorly to beta diversity matrices. However, the rare species have a stake in some communities with regard to beta diversity measures. Furthermore, the rare community does not appear to be a random assemblage given similar key groups and community patterns like those of the abundant community. The pronounced endemism of rare species supports the idea that the environment is selective among the rare population, too. Still, the largely unknown taxonomy of the rare methanogens highlights the necessary effort for improving the taxonomic identification through both culture-dependent and independent approaches. In the NGS era, large throughput allows for big and high-resolution data sets, the rare taxa can finally be taken into account for extending our insight into microbial occupation of niche spaces, ecological categories/strategies, community backup system and the diversity–function relationship. This knowledge will be valuable to offer additional information from those defined by phylogeny, taxonomy or functional capacity, and also to develop predictive models of microbial community resistance and response to environmental changes.

## SUPPLEMENTARY DATA

Supplementary data are available at *FEMSEC* online.

Supplemental material Supplementary data are available at *FEMSEC* online.

## References

[bib1] Angel R, Claus P, Conrad R Methanogenic archaea are globally ubiquitous in aerated soils and become active under wet anoxic conditions. ISME J 2012;6:847–62. 22071343 10.1038/ismej.2011.141 PMC3309352

[bib2] Bachy C, Worden AZ Microbial ecology: finding structure in the rare biosphere. Curr Biol 2014;24:R315–7. 24735853 10.1016/j.cub.2014.03.029

[bib3] Bagchi S, Tellez BG, Rao HA Diversity and dynamics of dominant and rare bacterial taxa in replicate sequencing batch reactors operated under different solids retention time. Appl Microbiol Biot 2015;99:2361–70. 10.1007/s00253-014-6134-4 25326778

[bib4] Baker FB Stability of two hierarchical grouping techniques case 1: Sensitivity to data errors. J Am Stat Assoc 1974; 69:440–5.

[bib5] Beck J, Holloway JD, Schwanghart A Undersampling and the measurement of beta diversity. Methods Ecol Evol 2013;4:370–82.

[bib6] Caron DA, Countway PD Hypotheses on the role of the protistan rare biosphere in a changing world. Aquat Microb Ecol 2009;57:227–38.

[bib7] Coveley S, Elshahed MS, Youssef NH Response of the rare biosphere to environmental stressors in a highly diverse ecosystem (Zodletone spring, OK, USA). PeerJ 2015;3:e1182. 26312178 10.7717/peerj.1182 PMC4548494

[bib8] Edgar RC, Haas BJ, Clemente JC UCHIME improves sensitivity and speed of chimera detection. Bioinformatics 2011;27:2194–200. 21700674 10.1093/bioinformatics/btr381 PMC3150044

[bib9] Fish JA, Chai B, Wang Q FunGene: the functional gene pipeline and repository. Front Microbiol 2013;4:291. 24101916 10.3389/fmicb.2013.00291 PMC3787254

[bib10] Frank-Fahle BA, Yergeau E, Greer CW Microbial functional potential and community composition in permafrost-affected soils of the NW Canadian Arctic. PLoS One 2014;9:e84761. 24416279 10.1371/journal.pone.0084761 PMC3885591

[bib11] Galand PE, Casamayor EO, Kirchman DL Ecology of the rare microbial biosphere of the Arctic Ocean. P Natl Acad Sci USA 2009;106:22427–32. 10.1073/pnas.0908284106 PMC2796907 20018741

[bib12] Galili T dendextend: an R package for visualizing, adjusting and comparing trees of hierarchical clustering. Bioinformatics 2015;31:3718–20. 26209431 10.1093/bioinformatics/btv428 PMC4817050

[bib13] Giovannoni SJ, Stingl U Molecular diversity and ecology of microbial plankton. Nature 2005;437:343–8. 16163344 10.1038/nature04158

[bib14] Gobet A, Boer SI, Huse SM Diversity and dynamics of rare and of resident bacterial populations in coastal sands. ISME J 2012;6:542–53. 21975598 10.1038/ismej.2011.132 PMC3280144

[bib15] Hedlund B, Staley J Microbial endemism and biogeography. In: Bull A (ed) Microbial Diversity and Bioprospecting.Washington, DC: ASM Press, 2004, 225–31.

[bib16] Heip CHR, Herman PMJ, Soetaert K Indices of diversity and evenness. Océanis 1998;24:61–87.

[bib17] Hewson I, Poretsky RS, Beinart RA In situ transcriptomic analysis of the globally important keystone N_2_-fixing taxon *Crocosphaera watsonii*. ISME J 2009;3:618–31. 19225552 10.1038/ismej.2009.8

[bib18] Hugoni M, Taib N, Debroas D Structure of the rare archaeal biosphere and seasonal dynamics of active ecotypes in surface coastal waters. P Natl Acad Sci USA 2013;110:6004–9. 10.1073/pnas.1216863110 PMC3625260 23536290

[bib19] Jousset A, Schmid B, Scheu S Genotypic richness and dissimilarity opposingly affect ecosystem functioning. Ecol Lett 2011;14:537–45. 21435139 10.1111/j.1461-0248.2011.01613.x

[bib20] Kleindienst S, Grim S, Sogin M Diverse, rare microbial taxa responded to the Deepwater Horizon deep-sea hydrocarbon plume. ISME J 2016;10:400–15. 26230048 10.1038/ismej.2015.121 PMC4737931

[bib20a] Liebner S, Ganzert L, Kiss A Shifts in methanogenic community composition and methane fluxes along the degradation of discontinuous permafrost. Front Microbiol 2015;6:356. 26029170 10.3389/fmicb.2015.00356 PMC4428212

[bib21] Logares R, Lindstrom ES, Langenheder S Biogeography of bacterial communities exposed to progressive long-term environmental change. ISME J 2013;7:937–48. 23254515 10.1038/ismej.2012.168 PMC3635229

[bib22] Loreau M, Hector A Partitioning selection and complementarity in biodiversity experiments. Nature 2001;412:72–76. 11452308 10.1038/35083573

[bib23] Luton PE, Wayne JM, Sharp RJ The *mcrA* gene as an alternative to 16S rRNA in the phylogenetic analysis of methanogen populations in landfill. Microbiology 2002;148:3521–30. 12427943 10.1099/00221287-148-11-3521

[bib24] Lynch MDJ, Bartram AK, Neufeld JD Targeted recovery of novel phylogenetic diversity from next-generation sequence data. ISME J 2012;6:2067–77. 22791239 10.1038/ismej.2012.50 PMC3475379

[bib25] Lynch MDJ, Neufeld JD Ecology and exploration of the rare biosphere. Nat Rev Microbiol 2015;13:217–29. 25730701 10.1038/nrmicro3400

[bib26] McGrady-Steed J, Harris PM, Morin PJ Biodiversity regulates ecosystem predictability. Nature 1997;390:162–5.

[bib27] McMurdie PJ, Holmes S phyloseq: an R package for reproducible interactive analysis and graphics of microbiome census data. PLoS One 2013;8:e61217. 23630581 10.1371/journal.pone.0061217 PMC3632530

[bib28] McMurdie PJ, Holmes S Waste not, want not: why rarefying microbiome data is inadmissible. PLoS Comp Biol 2014;10:e1003531. 10.1371/journal.pcbi.1003531 PMC3974642 24699258

[bib29] Nazaries L, Murrell JC, Millard P Methane, microbes and models: fundamental understanding of the soil methane cycle for future predictions. Environ Microbiol 2013;15:2395–417. 23718889 10.1111/1462-2920.12149

[bib30] Oksanen J, Blanchet FG, Kindt R vegan: Community Ecology Package. R package version 2.0-7, 2013. http://CRAN.R-project.org/package=vegan (8 October 2017, date last accessed).

[bib31] Pedros-Alio C Dipping into the rare biosphere. Science 2007;315:192–3. 17218512 10.1126/science.1135933

[bib32] Pedros-Alio C The rare bacterial biosphere. Ann Rev Mar Sci 2012;4:449–66. 10.1146/annurev-marine-120710-100948 22457983

[bib33] Pester M, Bittner N, Deevong P A ‘rare biosphere’ microorganism contributes to sulfate reduction in a peatland. ISME J 2010;4:1591–602. 20535221 10.1038/ismej.2010.75 PMC4499578

[bib34] Philippot L, Spor A, Henault C Loss in microbial diversity affects nitrogen cycling in soil. ISME J 2013;7:1609–19. 23466702 10.1038/ismej.2013.34 PMC3721106

[bib35] Pommier T, CanbÄCk B, Riemann L Global patterns of diversity and community structure in marine bacterioplankton. Mol Ecol 2007;16:867–80. 17284217 10.1111/j.1365-294X.2006.03189.x

[bib36] Reid A, Buckley M The Rare Biosphere.Washington, DC: American Academy of Microbiology, 2011, 32.

[bib37] Schloss PD, Westcott SL, Ryabin T Introducing mothur: open-source, platform-independent, community-supported software for describing and comparing microbial communities. Appl Environ Microb 2009;75:7537–41. 10.1128/AEM.01541-09 PMC2786419 19801464

[bib38] Shade A, Jones SE, Caporaso JG Conditionally rare taxa disproportionately contribute to temporal changes in microbial diversity. Mbio 2014;5:e01371–01314. 25028427 10.1128/mBio.01371-14 PMC4161262

[bib39] Sogin ML, Morrison HG, Huber JA Microbial diversity in the deep sea and the underexplored “rare biosphere”. P Natl Acad Sci USA 2006;103:12115–20. 10.1073/pnas.0605127103 PMC1524930 16880384

[bib40] Souza V, Eguiarte LE, Siefert J Microbial endemism: does phosphorus limitation enhance speciation? Nat Rev Microbiol 2008;6:559–64. 18521074 10.1038/nrmicro1917

[bib41] Steinberg LM, Regan JM Phylogenetic comparison of the methanogenic communities from an acidic, oligotrophic fen and an anaerobic digester treating municipal wastewater sludge. Appl Environ Microb 2008;74:6663–71. 10.1128/AEM.00553-08 PMC2576706 18776026

[bib42] Szekely AJ, Langenheder S The importance of species sorting differs between habitat generalists and specialists in bacterial communities. FEMS Microbiol Ecol 2014;87:102–12. 23991811 10.1111/1574-6941.12195

[bib43] Tilman D The ecological consequences of changes in biodiversity: A search for general principles. Ecology 1999;80:1455–74.

[bib44] Vergin KL, Done B, Carlson CA Spatiotemporal distributions of rare bacterioplankton populations indicate adaptive strategies in the oligotrophic ocean. Aquat Microb Ecol 2013;71:1–13.

[bib45] Wang Q, Quensen JF, Fish JA Ecological patterns of *nifH* genes in four terrestrial climatic zones explored with targeted metagenomics using FrameBot, a new informatics tool. Mbio 2013;4:e00592–13. 24045641 10.1128/mBio.00592-13 PMC3781835

[bib46] Wei T, Simko V Corrplot: Visualization of a Correlation Matrix. R package version 0.77 https://CRAN.R-project.org/package=corrplot 2016.

[bib47] Wen X, Yang S, Horn F Global biogeographic analysis of methanogenic archaea identifies community-shaping environmental factors of natural environments. Front Microbiol 2017;8:1339. 10.3389/fmicb.2017.01339 PMC5513909 28769904

[bib48] Wickham H ggplot2: Elegant Graphics for Data Analysis. New York: Springer, 2009.

[bib49] Yang S, Liebner S, Alawi M Taxonomic database and cut-off value for processing *mcrA* gene 454 pyrosequencing data by MOTHUR. J Microbiol Methods 2014;103:3–5. 24858450 10.1016/j.mimet.2014.05.006

[bib50] Yang S, Liebner S, Winkel M In-depth analysis of core methanogenic communities from high elevation permafrost-affected wetlands. Soil Biol Biochem 2017;111:66–77.

[bib51] Youssef NH, Couger MB, Elshahed MS Fine-scale bacterial beta diversity within a complex ecosystem (Zodletone Spring, OK, USA): the role of the rare biosphere. PLoS One 2010;5:e12414. 20865128 10.1371/journal.pone.0012414 PMC2932559

[bib52] Zavaleta ES, Hulvey KB Realistic variation in species composition affects grassland production, resource use and invasion resistance. Plant Ecol 2007;188:39–51.

[bib53] Zengler K, Toledo G, Rappe M Cultivating the uncultured. P Natl Acad Sci USA 2002;99:15681–6. 10.1073/pnas.252630999 PMC137776 12438682

