# Supplementary material for: Community structure of rare methanogenic archaea: insight from a single functional group
Source: FEMS Microbiol Ecol. 2017 Oct 3;93(11):fix126. doi: 10.1093/femsec/fix126 (PMC5812523; doi:10.1093/femsec/fix126)
Supplement: Supplemental material — Supplementary data are available at FEMSEC online. [file fix126_supp.docx]

**Supplementary material to ‘Community structure of rare methanogenic archaea: insight from a single functional group’**

Sizhong Yang^1, 2^, Matthias Winkel^1^, Dirk Wagner^1^, Susanne Liebner^1^

^1^ *GFZ German Research Center for Geosciences, Helmholtz Centre Potsdam, Section 5.3 Geomicrobiology, Telegrafenberg, 14473 Potsdam, Germany*

^2^ *State Key Laboratory of Frozen Soils Engineering, Northwest Institute of Eco-Environmental and Resources, Chinese Academy of Sciences, Lanzhou 730000, China*

This supplementary material includes Figure S1, S2 and S3, Table S1, S2 and S3.

**Figure S1** Rarefaction curve (a) and correlation between the sequence numbers and OTU richness (b), suggest that the sequencing depth did not substantially influence the diversity measures.


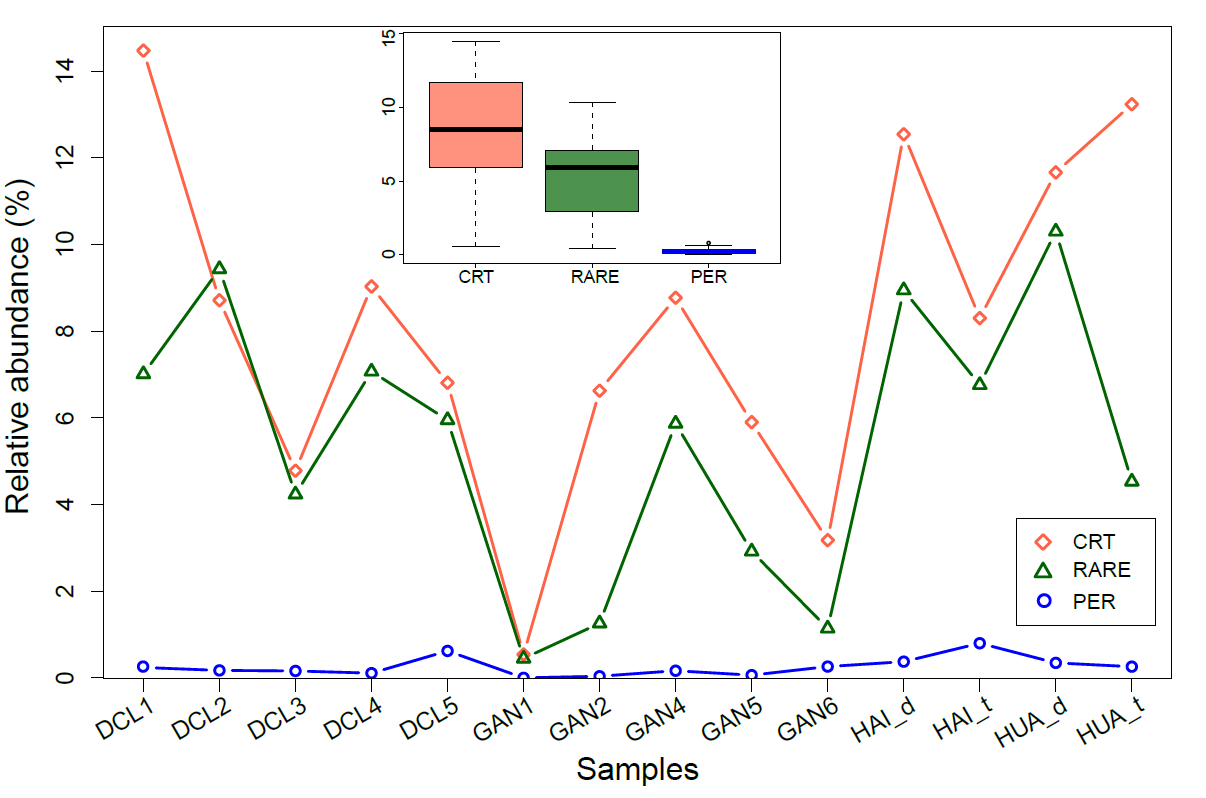


**Figure S2** Abundance distribution patterns of the rare biosphere given by rare clusters and by samples. The line plot shows the fluctuation of each rare cluster by samples, and the inlet box plot displays the variations of abundance for each cluster. The ‘CRT’ and ‘PER’ represent the ‘conditionally rare taxa’ and ‘permanently rare taxa’, respectively. All the other rare ones were assigned as ‘RARE’. DCL: Donggi Cona Lake region; HAI: Haibei Station; HUA: Huashixia and GAN: Gande. The numeric labels after the site abbreviations indicate the different samples.

**Figure S3** Bubble plot illustrating the variations of different taxa at the genus-level in the rare biosphere over different samples. The taxonomic classification was performed based on the phylogenetic neighbor joining tree calculated in ARB (not shown). The methanogenic taxa at the vertical axis are ordered according to their average relative abundance at the genus level with the most abundant taxa at the top and the least abundant taxa at the bottom. DCL: Donggi Cona Lake region; HAI: Haibei Station; HUA: Huashixia and GAN: Gande. The numeric labels after the site abbreviations indicate the different samples.

**Figure S3** Bubble plot illustrating the variations of different taxa at the genus-level in the rare biosphere over different samples. The taxonomic classification was performed based on the phylogenetic neighbor joining tree calculated in ARB (not shown). The taxonomy is shown for the genus level. If an assignment to the genus level was not possible the next higher assignable taxonomical level was used. The methanogenic taxa at the vertical axis are ordered according to their average relative abundance with the most abundant taxa at the top and the least abundant taxa at the bottom. DCL: Donggi Cona Lake region; HAI: Haibei Station; HUA: Huashixia and GAN: Gande. The numeric labels after the site abbreviations indicate the different samples.

**Table S1**. Relative abundance of the abundant lineages (adopted from Yang et al., 2017)

| Lineages | Mean relative abundance (%) |
| --- | --- |
| *Methanoregula* | 34.7 |
| *Methanomassiliicoccus* | 18.7 |
| *Methanosarcina* | 14.5 |
| *Methanosaeta* | 6.7 |
| *Methanocella* | 6.2 |
| *Methanolobus* | 3.3 |
| *Methanobacterium* | 2.4 |

**Table S2**. Summary table of rare taxa showing the ratio between the maximum and minimum relative abundances and classification of different types of rarity. CRT: conditionally rare taxa; PER: permanently rare taxa; RARE: all the other rare taxa excluding CRT and PER. Max Relabund: maximum relative abundance; Min Relabund: minimum relative abundance.

| OTUs | Max  Relabund | Min  Relbund | ratio | Type | Genus |
| --- | --- | --- | --- | --- | --- |
| OTU020 | 8.9368 | 0.0067 | 1333.85 | CRT | Methanoregula |
| OTU022 | 2.6321 | 0.0334 | 78.81 | RARE | Methanobacterium |
| OTU025 | 4.4401 | 0.0138 | 321.75 | CRT | Methanobacterium |
| OTU026 | 2.7297 | 0.0067 | 407.42 | CRT | Methanothermobacter |
| OTU028 | 4.3301 | 0.0057 | 759.67 | CRT | Methanosarcina |
| OTU029 | 7.1036 | 0.0345 | 205.90 | CRT | Methanomethylovorans |
| OTU030 | 3.6916 | 0.0267 | 138.26 | CRT | Methanocella |
| OTU033 | 1.6062 | 0.0083 | 193.52 | CRT | Methanoregula |
| OTU034 | 1.6043 | 0.04 | 40.11 | CRT | Methanoregula |
| OTU035 | 1.4407 | 0.0133 | 108.32 | CRT | Methanomassiliicoccus |
| OTU036 | 2.6899 | 0.0275 | 97.81 | RARE | Methanocella |
| OTU037 | 3.7946 | 0.0056 | 677.61 | CRT | Methanocella |
| OTU038 | 1.686 | 0.0333 | 50.63 | RARE | uncultured_Methanosaetaceae_environ_samples |
| OTU039 | 1.2488 | 0.04 | 31.22 | RARE | Methanospirillum |
| OTU040 | 1.6512 | 0.0167 | 98.87 | RARE | Methanomassiliicoccus |
| OTU041 | 2.312 | 0.0345 | 67.01 | RARE | Methanoregula |
| OTU042 | 1.6401 | 0.0345 | 47.54 | RARE | Methanobacterium |
| OTU043 | 1.5849 | 0.0133 | 119.17 | CRT | Methanosaeta |
| OTU044 | 1.0032 | 0.0133 | 75.43 | RARE | Methanosarcina |
| OTU045 | 1.0515 | 0.0111 | 94.73 | RARE | Methanosaeta |
| OTU046 | 2.1735 | 0.0248 | 87.64 | RARE | Methanobacterium |
| OTU047 | 0.9233 | 0.0533 | 17.32 | RARE | Methanosaeta |
| OTU048 | 0.7003 | 0.0256 | 27.36 | RARE | Methanosaeta |
| OTU049 | 1.449 | 0.0138 | 105.00 | CRT | Methanoregula |
| OTU050 | 2.2943 | 0.0164 | 139.90 | CRT | Methanoregula |
| OTU051 | 0.8311 | 0.0667 | 12.46 | RARE | Methanoregula |
| OTU052 | 1.303 | 0.0138 | 94.42 | RARE | uncultured_Methanosaetaceae_environ_samples |
| OTU053 | 1.0695 | 0.0057 | 187.63 | CRT | Methanoregula |
| OTU054 | 1.2248 | 0.148 | 8.28 | RARE | unclassified_Methanosaetaceae |
| OTU055 | 0.8763 | 0.0057 | 153.74 | CRT | Methanobacterium |
| OTU056 | 0.698 | 0.0076 | 91.84 | RARE | Methanobacterium |
| OTU057 | 1.2765 | 0.0492 | 25.95 | RARE | Methanosaeta |
| OTU058 | 0.7749 | 0.0057 | 135.95 | CRT | Methanoculleus |
| OTU059 | 0.4731 | 0.0057 | 83.00 | RARE | Methanoregula |
| OTU060 | 0.8245 | 0.0055 | 149.91 | CRT | Methanoregulac |
| OTU061 | 0.8121 | 0.1035 | 7.85 | RARE | unclassified_Methanosaetaceae |
| OTU062 | 0.4818 | 0.0133 | 36.23 | RARE | Methanobacterium |
| OTU063 | 0.339 | 0.0068 | 49.85 | RARE | Methanosarcina |
| OTU064 | 0.4724 | 0.0057 | 82.88 | RARE | Methanoregula |
| OTU065 | 0.5913 | 0.0056 | 105.59 | CRT | Methanosarcina |
| OTU066 | 0.2466 | 0.0109 | 22.62 | RARE | Methanoregula |
| OTU067 | 0.4547 | 0 | - | PER | Methanobacterium |
| OTU068 | 0.2027 | 0.0057 | 35.56 | RARE | Methanoculleus |
| OTU069 | 0.4129 | 0.0991 | 4.17 | PER | Methanoculleus |
| OTU070 | 0.2779 | 0.0248 | 11.21 | RARE | Methanoregula |
| OTU071 | 0.1799 | 0.0055 | 32.71 | RARE | Methanosarcina |
| OTU072 | 0.109 | 0.0056 | 19.46 | RARE | Methanosphaerula |
| OTU073 | 0.223 | 0.0056 | 39.82 | RARE | Methanobrevibacter |
| OTU074 | 0.3104 | 0.0133 | 23.34 | RARE | Methanoregula |
| OTU075 | 0.0762 | 0.0219 | 3.48 | PER | Methanoregula |
| OTU076 | 0.0611 | 0.0109 | 5.61 | RARE | Methanomassiliicoccus |
| OTU077 | 0.1281 | 0.0076 | 16.86 | RARE | unclassified_Euryarchaeota |
| OTU078 | 0.0869 | 0.0067 | 12.97 | RARE | Methanoregula |
| OTU079 | 0.185 | 0.0138 | 13.41 | RARE | Methanoregula |
| OTU080 | 0.2057 | 0.0083 | 24.78 | RARE | unclassified_Methanosaetaceae |
| OTU081 | 0.2001 | 0 | - | PER | unclassified_Euryarchaeota |
| OTU082 | 0.1147 | 0.0055 | 20.85 | RARE | unclassified_Methanosarcinaceae |
| OTU083 | 0.069 | 0.0111 | 6.22 | RARE | unclassified_Methanosaetaceae |
| OTU084 | 0.0768 | 0.0743 | 1.03 | PER | Methanolobus |
| OTU085 | 0.0863 | 0.0138 | 6.25 | RARE | unclassified_Methanosaetaceae |
| OTU086 | 0.1524 | 0 | - | PER | uncultured_methanogen_RS-MCR04 |
| OTU087 | 0.0811 | 0.0055 | 14.75 | RARE | unclassified_Methanosaetaceae |
| OTU088 | 0.0833 | 0.0056 | 14.88 | RARE | unclassified_Methanoregulaceae |
| OTU089 | 0.0621 | 0.0057 | 10.89 | RARE | unclassified_Methanosaetaceae |
| OTU090 | 0.069 | 0.0055 | 12.55 | RARE | Methanoregula |
| OTU091 | 0.1101 | 0.0057 | 19.32 | RARE | unclassified_Methanosaetaceae |
| OTU092 | 0.0656 | 0 | - | PER | Methanomassiliicoccus |
| OTU093 | 0.0551 | 0.0057 | 9.67 | RARE | unclassified_Methanosaetaceae |
| OTU094 | 0.0551 | 0.0083 | 6.64 | RARE | unclassified_Methanosaetaceae |
| OTU095 | 0.0692 | 0 | - | PER | unclassified_Euryarchaeota |
| OTU096 | 0.0333 | 0.0115 | 2.90 | PER | Methanoregula |
| OTU097 | 0.0459 | 0 | - | PER | Methanoregula |
| OTU098 | 0.0278 | 0.0057 | 4.88 | PER | Methanoregula |
| OTU099 | 0.1035 | 0.0138 | 7.50 | RARE | Methanobrevibacter |
| OTU100 | 0.0333 | 0.0173 | 1.92 | PER | unclassified_Methanosaetaceae |
| OTU101 | 0.0333 | 0 | - | PER | Methanocella |
| OTU102 | 0.0275 | 0.0219 | 1.26 | PER | unclassified_Methanosaetaceae |
| OTU103 | 0.0688 | 0 | - | PER | Methanobrevibacter |
| OTU104 | 0.0167 | 0.0138 | 1.21 | PER | unclassified_Methanosaetaceae |
| OTU105 | 0.0222 | 0.0076 | 2.92 | PER | Methanoregula |
| OTU106 | 0.0219 | 0.0173 | 1.27 | PER | uncultured_Methanosaetaceae_environ_samples |
| OTU107 | 0.0497 | 0.0055 | 9.04 | RARE | Methanoregula |
| OTU108 | 0.0275 | 0.0152 | 1.81 | PER | Methanosaeta |
| OTU109 | 0.0551 | 0.0083 | 6.64 | RARE | Methanoregula |
| OTU110 | 0.0256 | 0 | - | PER | Methanomassiliicoccus |
| OTU111 | 0.0256 | 0 | - | PER | unclassified_Methanothermus |
| OTU112 | 0.0518 | 0.0056 | 9.25 | RARE | Methanobacterium |
| OTU113 | 0.0277 | 0 | - | PER | unclassified_Methanoregulaceae |
| OTU114 | 0.0124 | 0.0055 | 2.25 | PER | unclassified_Methanoregulaceae |
| OTU115 | 0.0115 | 0.0064 | 1.80 | PER | unclassified_Methanosaetaceae |
| OTU116 | 0.0275 | 0.0056 | 4.91 | PER | unclassified_Methanosaetaceae |
| OTU117 | 0.0138 | 0.0083 | 1.66 | PER | unclassified_Methanosaetaceae |
| OTU118 | 0.0115 | 0.0068 | 1.69 | PER | unclassified_Methanocorpusculaceae |
| OTU119 | 0.0208 | 0 | - | PER | unclassified_Methanomicrobiaceae |
| OTU120 | 0.0518 | 0 | - | PER | Methanobacterium |
| OTU121 | 0.0135 | 0.0069 | 1.96 | PER | unclassified_Methanosarcinaceae |
| OTU122 | 0.0413 | 0 | - | PER | unclassified_Methanomicrobia |
| OTU123 | 0.0167 | 0 | - | PER | unclassified_Methanoregulaceae |
| OTU124 | 0.0115 | 0 | - | PER | Methanoregula |
| OTU125 | 0.0111 | 0 | - | PER | Methanoregula |
| OTU126 | 0.0109 | 0 | - | PER | unclassified_Methanoregulaceae |
| OTU127 | 0.0138 | 0.0057 | 2.42 | PER | unclassified_Methanolinea |
| OTU128 | 0.0111 | 0 | - | PER | unclassified_Methanomicrobiales |
| OTU129 | 0.0111 | 0 | - | PER | Methanomassiliicoccus |
| OTU130 | 0.0115 | 0 | - | PER | unclassified_Methanosaetaceae |
| OTU131 | 0.0057 | 0.0056 | 1.02 | PER | unclassified_Methanosarcinaceae |
| OTU132 | 0.0345 | 0 | - | PER | Methanosphaera |
| OTU133 | 0.0124 | 0.0056 | 2.21 | PER | Methanoregula |
| OTU134 | 0.0111 | 0 | - | PER | Methanospirillum |
| OTU135 | 0.0152 | 0 | - | PER | unclassified_Methanosaetaceae |
| OTU136 | 0.0055 | 0 | - | PER | unclassified_Methanocellaceae |
| OTU137 | 0.0067 | 0 | - | PER | Methanoregula |
| OTU138 | 0.0057 | 0 | - | PER | unclassified_Methanomicrobiaceae |
| OTU139 | 0.0064 | 0 | - | PER | unclassified_Euryarchaeota |
| OTU140 | 0.0173 | 0 | - | PER | uncultured_Methanosaetaceae_environ_samples |
| OTU141 | 0.0064 | 0 | - | PER | Methanomassiliicoccus |
| OTU142 | 0.0083 | 0 | - | PER | Methanomassiliicoccus |
| OTU143 | 0.0124 | 0 | - | PER | uncultured_Methanosaetaceae_environ_samples |
| OTU144 | 0.0057 | 0 | - | PER | Methanomethylovorans |
| OTU145 | 0.0067 | 0 | - | PER | Methanoregula |
| OTU146 | 0.0138 | 0 | - | PER | uncultured_Methanosaetaceae_environ_samples |
| OTU147 | 0.0057 | 0 | - | PER | Methanomassiliicoccus |
| OTU148 | 0.0056 | 0 | - | PER | Methanomassiliicoccus |
| OTU149 | 0.0138 | 0 | - | PER | unclassified_Methanosaetaceae |
| OTU150 | 0.0055 | 0 | - | PER | unclassified_Methanoregulaceae |
| OTU151 | 0.0067 | 0 | - | PER | Methanomassiliicoccus |
| OTU152 | 0.0056 | 0 | - | PER | Methanoculleus |
| OTU153 | 0.0056 | 0 | - | PER | unclassified_Methanosarcinaceae |
| OTU154 | 0.0138 | 0 | - | PER | unclassified_Methanosaetaceae |
| OTU155 | 0.0069 | 0 | - | PER | unclassified_Methanosarcinaceae |
| OTU156 | 0.0124 | 0 | - | PER | unclassified_Methanomicrobiales |
| OTU157 | 0.0068 | 0 | - | PER | Methanomassiliicoccus |
| OTU158 | 0.0069 | 0 | - | PER | Methanomassiliicoccus |
| OTU159 | 0.0056 | 0 | - | PER | unclassified_Methanoculleus |
| OTU160 | 0.0057 | 0 | - | PER | uncultured_Methanosaetaceae_environ_samples |
| OTU161 | 0.0138 | 0 | - | PER | uncultured_Methanosaetaceae_environ_samples |
| OTU162 | 0.037 | 0 | - | PER | uncultured_Methanosaetaceae_environ_samples |
| OTU163 | 0.0138 | 0 | - | PER | unclassified_Methanoregulaceae |
| OTU164 | 0.0069 | 0 | - | PER | Methanomassiliicoccus |
| OTU165 | 0.0083 | 0 | - | PER | Methanomassiliicoccus |
| OTU166 | 0.0173 | 0 | - | PER | unclassified_Methanobacteriaceae |
| OTU167 | 0.0068 | 0 | - | PER | unclassified_Methanosarcinaceae |
| OTU168 | 0.0057 | 0 | - | PER | Methanomassiliicoccus |
| OTU169 | 0.0057 | 0 | - | PER | unclassified_Methanomassiliicoccaceae |
| OTU170 | 0.0064 | 0 | - | PER | unclassified_Methanosaetaceae |
| OTU171 | 0.0057 | 0 | - | PER | Methanoregula |
| OTU172 | 0.0055 | 0 | - | PER | Methanoregula |
| OTU173 | 0.0056 | 0 | - | PER | Methanomassiliicoccus |
| OTU174 | 0.0067 | 0 | - | PER | uncultured_methanogen_RS-MCR04 |
| OTU175 | 0.0076 | 0 | - | PER | unclassified_Methanosaetaceae |

**Table S3**. Summary of contribution of conditionally rare taxa on the total community Bray-Curtis (BC) dissimilarity (multiplied by 100 will be percentage). The labels ‘rare2total BC’ and ‘CRT2total BC’ stand for the ‘whole rare taxa to the total Bray-Curtis dissimilarity’ and ‘CRT to the total Bray-Curtis dissimilarity’, respectively.

| Statistic | rare2total BC | CRT2total BC |
| --- | --- | --- |
| Minimum | 0.02255 | 0.01325 |
| 1st Quantile | 0.10455 | 0.05849 |
| Median | 0.13942 | 0.07846 |
| Mean | 0.14466 | 0.08671 |
| 3rd Quantile | 0.17584 | 0.11134 |
| Maximum | 0.35597 | 0.24694 |

**Reference**

Yang S, Liebner S, Winkel M, Alawi M, Horn F, Dörfer C et al (2017). In-depth analysis of core methanogenic communities from high elevation permafrost-affected wetlands. Soil Biology and Biochemistry 111: 66-77.
